# Relationship between Humoral Response in COVID-19 and Seasonal Influenza Vaccination

**DOI:** 10.3390/vaccines10101621

**Published:** 2022-09-27

**Authors:** Barbara Poniedziałek, Ewelina Hallmann, Dominika Sikora, Karol Szymański, Katarzyna Kondratiuk, Jakub Żurawski, Piotr Rzymski, Lidia Brydak

**Affiliations:** 1 Department of Environmental Medicine, Poznań University of Medical Sciences, 60-806 Poznan, Poland; 2 Department of Influenza Research, National Influenza Center at the National Institute of Public Health NIH—National Research Institute in Warsaw, Chocimska St. 24, 00-791 Warsaw, Poland; 3 Doctoral School, Poznan University of Medical Sciences, Fredry St. 10, 61-701 Poznan, Poland; 4 Department of Immunobiology, Poznan University of Medical Sciences, 60-806 Poznan, Poland; 5 Integrated Science Association (ISA), Universal Scientific Education and Research Network (USERN), 60-806 Poznan, Poland

**Keywords:** heterologous protection, trained immunity, adaptive immunity, immunology, SARS-CoV-2, pandemic

## Abstract

There is evidence that vaccination against seasonal influenza can improve innate immune responses to COVID-19 and decrease disease severity. However, less is known about whether it could also impact the humoral immunity in SARS-CoV-2 infected patients. The present study aimed to compare the SARS-CoV-2 specific humoral responses (IgG antibodies against nucleocapsid; anti-N, receptor binding domain; anti-RBD, subunit S2; anti-S2, and envelope protein; anti-E) between non-hospitalized, COVID-19 unvaccinated, and mild COVID-19 convalescent patients who were and were not vaccinated against influenza during the 2019/2020 epidemic season (*n* = 489 and *n* = 292, respectively). The influenza-vaccinated group had significantly higher frequency and titers of anti-N antibodies (75 vs. 66%; mean 559 vs. 520 U/mL) and anti-RBD antibodies (85 vs. 76%; mean 580 vs. 540 U/mL). The prevalence and concentrations of anti-S2 and anti-E antibodies did not differ between groups (40–43%; mean 370–375 U/mL and 1.4–1.7%; mean 261–294 U/mL) and were significantly lower compared to those of anti-RBD and anti-N. In both groups, age, comorbidities, and gender did not affect the prevalence and concentrations of studied antibodies. The results indicate that influenza vaccination can improve serum antibody levels produced in response to SARS-CoV-2 infection.

## 1. Introduction

A broad range of factors can affect the host immune response to viral infection, including the pathogen’s immunogenicity, the disease’s clinical course, human age, sex, and health status [1,2,3]. During the pandemic of coronavirus disease 2019 (COVID-19), increasing attention has been given to the cross-protective effects of different vaccinations. As demonstrated by selected epidemiological studies, individuals vaccinated against influenza had lower odds of SARS-CoV-2 infection, hospitalization, need for mechanical ventilation, and death due to COVID-19 [4,5,6]. The data also demonstrate that the bacillus Calmette−Guérin (BCG) vaccine against tuberculosis can confer protection against other infectious diseases, including influenza staphylococci and yellow fever [7,8,9]. This phenomenon has been attributed to the so-called “trained immunity”, a process of epigenetic reprogramming of transcriptional pathways induced by infections and vaccinations that ultimately allows the innate immune system to exhibit adaptive characteristics [10,11].

However, there is also initial evidence that previous vaccinations against other respiratory diseases could improve the humoral response to the COVID-19 vaccine. In one study, individuals receiving concomitant influenza and pneumococcal or only influenza vaccination revealed significantly increased micro-neutralization titers after administration of the BNT162b2 vaccine (BioNTech/Pfizer, Germany, Mainz/New York, NY, USA) compared to those not vaccinated against influenza/pneumococcal disease [12]. Another study recently confirmed this finding, demonstrating higher titers of antibodies against the SARS-CoV-2 receptor binding domain following BNT162b2 vaccination in healthcare workers who previously received the seasonal influenza vaccine [13]. The exact molecular mechanisms behind this effect are yet to be elucidated.

The first investigations of the humoral response to hemagglutinins of the influenza virus during the COVID-19 pandemic [14] provided the passage for further studies evaluating whether vaccination against seasonal influenza could also impact the humoral immunity in SARS-CoV-2 infected patients is less known. Therefore, the present study aimed to compare the SARS-CoV-2 specific humoral responses between non-hospitalized, COVID-19 unvaccinated, and mild COVID-19 convalescent patients who were and were not previously vaccinated against influenza during the 2019/2020 epidemic season. To this end, the prevalence and concentrations of four IgG antibodies specific to SARS-CoV-2 were evaluated in both groups.

## 2. Materials and Methods

### 2.1. Patients and Serum Samples

All serum samples were purchased in 2020 from the Regional Blood Donation and Blood Treatment Centers in Poland from units located in 8 voivodeships in the following cities: Białystok, Warsaw, Radom, Racibórz, Kalisz, Bydgoszcz, Łódź, Szczecin, and Wrocław. All samples were collected between September and December 2020 from SARS-CoV-2 infected patients (confirmed by RT-PCR) 1 month (+/− 2 weeks) after the resolution of symptoms/end of the isolation period. This period was dominated by infections with Nextstrain clades 20A, 20B, and 20C [15], which did not reveal major differences in clinical outcomes [16,17]. In total, we purchased 659 serum samples from individuals vaccinated against influenza during 2019/2020 epidemic season and 659 serum samples from unvaccinated persons. All influenza-vaccinated individuals received the vaccine in the recommended period between September and December 2019, approximately one year prior to infection with SARS-CoV-2. The patient’s age, gender, comorbidities (present or not), and COVID-19 severity were collected for all samples. The frozen samples were transported frozen to the Department of Influenza Research, National Influenza Centre in National Institute of Public Health—National Research Institute. The research project was approved by the Bioethical Committee of the Institute of Public Health—National Research Institute (approval no. 4/2020; date of approval: 6 August 2020) and the Bioethics Committee at Poznan University of Medical Sciences (approval no. 429/22; date of approval: 11 May 2022). Considering that severity of SARS-CoV-2 infection can significantly influence the humoral responses [18,19], individuals who underwent mild COVID-19, not requiring hospitalization, were selected for this analysis. In total, 781 sera samples were analyzed, with 292 originating from individuals not vaccinated against influenza and 489 from those vaccinated in the 2019/2020 epidemic season. As the samples originated from 2020, all individuals were not vaccinated against COVID-19.

### 2.2. Determination of Anti-SARS-CoV-2-Specific IgG Antibodies

The collected serum samples were tested using the CE-IVD certified Microblot-Array COVID-19 IgG assay (TestLine Clinical Diagnostics, Brno, Czech Republic) for the presence and titer of the specific SARS-CoV-2 IgG antibodies against the receptor binding domain of the spike protein (anti-RBD), S2 subunit of the spike protein (anti-S2), nucleocapsid protein (anti-N), and envelope protein (anti-E). In this assay, recombinant and purified native antigens are immobilized on specific spots of nitrocellulose membrane fixed at the bottom of the microplate well [20]. The concentrations for all four antibodies were given as U/mL and interpreted as positive if above 210 U/mL.

### 2.3. Statistical Analyses

Data were analyzed with Statistica v.13.3 (StatSoft Inc., Tulsa, OK, USA). Because no assumption of Gaussian distribution was met (Shapiro–Wilk’s test, *p* < 0.05), a non-parametric Mann–Whitney U test was employed to compare groups vaccinated and unvaccinated against influenza. Comparison of titers of different antibodies was performed with Kruskal–Wallis ANOVA using Dunn’s test as a post hoc method. Spearman’s rank coefficient was used to assess the relationship between concentrations of different antibodies. The prevalence of antibodies in influenza vaccinated and unvaccinated were compared with Pearson’s χ^2^ test. When *p* < 0.05, differences were deemed statistically significant.

## 3. Results

### 3.1. Demographic Characteristics

Serum samples collected from 781 mild COVID-19 convalescent patients were analyzed, among whom 62.6% were vaccinated against influenza in the 2019/2020 infection season. Groups of patients vaccinated and unvaccinated against influenza did not differ in age and gender, but the former was represented by a higher frequency of comorbidities (Table 1).

### 3.2. Prevalence of SARS-CoV-2-Specific IgG Antibodies

The prevalence of anti-N, anti-RBD, anti-S2, and anti-E IgG antibodies in the studied cohort was 71.3, 81.6, 41.7, and 1.5%, respectively (Table 2). In general, 12.7% had undetectable levels of any of the considered antibodies, 15.7% tested positive for one, 35.7% for two, 34.4% for three, and 1.4% for all four. Group vaccinated against influenza in the 2019/2020 season revealed a higher prevalence of anti-N (by 8.8%) and anti-RBD (by 8.4%) antibodies compared to those who did not receive such vaccination (Table 2). In both groups, the prevalence of any antibody was not differentiated by age ≥ 50 years, comorbidities (*p* > 0.05 in all cases, Pearson’s χ^2^ test), or between women and men (*p* > 0.05 in all cases, Mann–Whitney U test).

### 3.3. Titers of SARS-CoV-2-Specific IgG Antibodies

**Figure 1:**
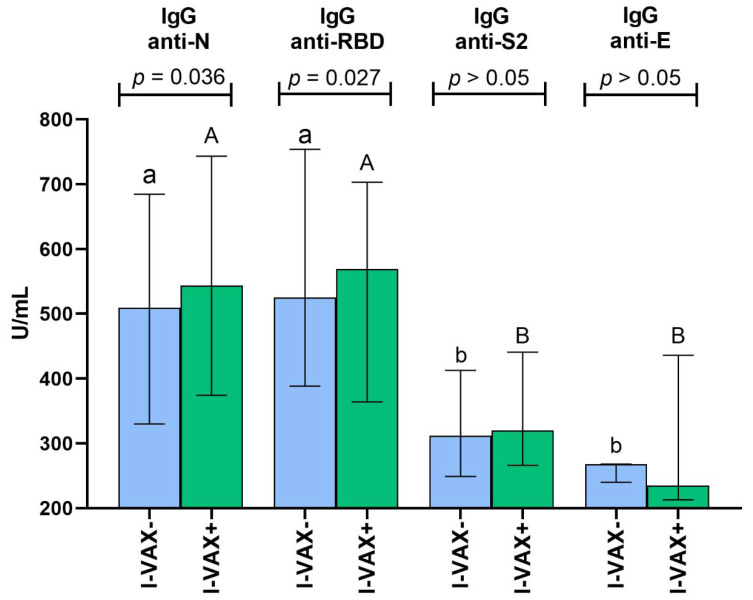
Serum titers (median and interquartile range) of IgG antibodies against SARS-CoV-2 nucleocapsid protein (anti-N), receptor binding domain of spike protein (anti-RBD), subunit S2 of spike protein (anti-S2), and envelope protein (anti-E) in mild COVID-19 convalescent individuals not vaccinated (I-VAX-) and vaccinated (I-VAX+) against seasonal influenza. The *p*-value refers to the difference between these groups examined with the Mann–Whitney U test. Different small letters (a, b) above bars indicate a significant difference between antibody concentrations within the I-VAX- group, while different capital letters (A, B) indicate it within the I-VAX+ group (Kruskal–Wallis ANOVA with Dunn’s post hoc test).

Generally, the serum concentrations of anti-N, anti-RBD, anti-S2, and anti-E IgG antibodies (mean ± SD) in all studied patients who tested positive for their presence were 545.8 ± 212.6, 566.0 ± 217.7, 373.2 ± 165.3, and 280.3 ± 78.8 U/mL, respectively. Group vaccinated against seasonal influenza revealed significantly higher concentrations of anti-N and anti-RBD antibodies than those who did not receive the influenza vaccine; the difference in means was 39.5 (7.6%) and 40.0 (7.4%) U/mL, respectively (Figure 1). Within both subgroups, titers of anti-N and anti-RBD antibodies were higher than that of anti-S2 and anti-E (Figure 1).

In both groups, serum concentration of any antibody was not differentiated by age ≥ 50 years or comorbidities and did not differ between women and men (*p* > 0.05 in all cases, Mann–Whitney U test). The serum concentrations of anti-N were significantly correlated with anti-RBD and anti-S2 titers in both groups. Additionally, in individuals vaccinated against seasonal influenza, anti-RBD and anti-S2 concentrations were positively associated (Table 3).

## 4. Discussion

The present study demonstrated some beneficial relationship between seasonal influenza vaccination and humoral response in SARS-CoV-2 infection. Individuals who received the influenza vaccine during the 2019/2020 epidemic season revealed higher frequency and titers of anti-N and anti-RBD IgG antibodies. The increased levels of these antibodies can translate into better protection against reinfection or exert neutralization effects if the virus still replicates in tissues [21]. Although age, gender, and comorbidities were previously observed as potential factors influencing humoral responses in COVID-19 [22,23,24,25], this was not the case in the present cohort of patients who underwent mild disease. These findings add to the body of knowledge on the positive effects of influenza vaccination in COVID-19 [4,5,6,26,27].

Our results suggest that influenza vaccination may increase the strength of the adaptive response to other viral infections. Although the mechanisms behind this phenomenon are not known, it can be speculated that vaccination positively affects the production of interleukin-4 by T-helper 2 cells, leading to better clonal expansion of B cells and/or interleukin-5 and interleukin-6, which contribute to later phases of B-cell activation by driving their differentiation and supporting antibody production [28]. Moreover, it is suggested that influenza vaccination may induce innate immune training in myeloid cells by altering cytokine production through epigenetic changes [29,30,31]. It is plausible that such trained myeloid cells may also support humoral responses during SARS-CoV-2 infection. Further investigations are required to understand better the exact nature of immunological events in play and their role in the cross-protective effects of influenza vaccination against heterologous infection.

Compared to anti-RBD IgG antibodies, anti-N were less prevalent in the studied cohort (by 10.3%), as well as in subsets of individuals vaccinated (by 10.5%) and unvaccinated (by 10.9%) against seasonal influenza. This is in line with other studies, which also reported a lower prevalence of anti-N IgG antibodies compared to anti-RBD [32,33]. This is due to the different dynamics of these antibodies, from which anti-N are detected earlier and have a significantly lower half-life [33,34]. Moreover, a lower prevalence of anti-N antibodies is likely also due to the location of nucleocapsid protein inside the lipid bilayer envelope, which can blunt its recognition by immune cells [35,36]. In turn, less than 50% of analyzed serum samples were positive for anti-S2 IgG antibodies. Experimental vaccine research revealed that the S2 subunit of SARS-CoV-2 S protein, which has distinct domains involved in mediating viral fusion of viral envelope, can be similarly immunogenic as S1, which contains RBD and the N-terminal domain [37]. However, these observations relate to the immunogenicity comparison of different subunit vaccine candidates, whereas in the case of the virion, S2 is much less accessible for immune cell recognition and contains a lower number of predicted epitopes than S1 [38]. Similarly to our observations, other studies also reported a low prevalence of anti-S2 IgG antibodies. For example, an Italian serological study found that the prevalence of anti-S2 IgG antibodies in SARS-CoV-2 infected patients was 42% compared to 87% for anti-S1 and 93% for anti-RBD [39]. Notably, the S2 subunit is more conserved among coronaviruses than S1 [40], while anti-S2 antibodies can harbor Fc-dependent effect function [41] and reveal pan-betacoronavirus neutralization potencies [42,43,44]. Therefore, their presence can enhance the host’s antiviral humoral immunity. In our study, the prevalence of anti-S2 Igg antibodies in individuals vaccinated against influenza was only slightly and statistically insignificantly higher compared to unvaccinated patients (by 3.2%), while serum concentrations in both groups were similar. However, in the former subset of subjects, the anti-S2 titers were positively correlated with those of anti-RBD. Although the exact nature of this relationship remains unclear, it may suggest that vaccination against influenza could enhance the simultaneous recognition of S2 and RBD in some individuals.

We also found that influenza vaccination was not associated with a more frequent presence or higher serum levels of anti-E IgG antibodies. Moreover, these antibodies were very rare in the studied cohort, and their concentration was significantly lower than that of anti-N and anti-RBD. Other serological research also observed a very low or zero prevalence of anti-E IgG antibodies [20,45]. The envelope protein is the smallest structural protein of SARS-CoV-2 (length 75 amino acids) and has a low protrusion of its ectodomains that could be recognized as epitopes [35,46]. Although it is abundantly expressed inside the infected cell, only a small portion is incorporated into the virion envelope [47,48].

Our study has some limitations. Firstly, serum samples were collected before the emergence of SARS-CoV-2 variants of concern, such as Alpha, Delta, and Omicron, which may differ in clinical severity and antigenicity [17,49]. Secondly, due to the unavailability of data, the study did not include some patient characteristics, which may also influence humoral responses, e.g., body mass index, specific comorbidities, or the use of medications (prior to and during the SARS-CoV-2 infection). However, one should note that the studied individuals underwent mild COVID-19 and did not require hospitalization. Thus, it is unlikely they were ordered any specific anti-SARS-CoV-2 treatment that could affect humoral responses (e.g., glucocorticoid), as such treatment was not recommended at the time of our study (September–December 2020), while specific anti-SARS-CoV-2 medications were not available [50,51]. Further research is required to understand whether influenza vaccination could be associated with modified humoral response in asymptomatic and severe SARS-CoV-2 infections. Moreover, it is unknown whether influenza vaccination could also be associated with the response of other immunoglobulin classes that play an important role in SARS-CoV-2 infection, i.e., IgM and IgA [52]. The potential association between repeated influenza vaccination with humoral responses in COVID-19 also remains to be investigated since some data show that it may blunt immune reactions and lead to a decline in the effectiveness of influenza vaccines (although this phenomenon remains controversial, while the underlying mechanism is not clear) [53,54]. One should also bear in mind that our study did not investigate the function of anti-SARS-CoV-2 antibodies. Therefore, whether higher antibody concentrations found for influenza-vaccinated individuals would translate into better virus neutralization requires further research. However, it was demonstrated that the presence of antibodies, such as IgG anti-N, the prevalence of which was higher in individuals vaccinated against influenza, was associated with a substantially reduced risk of reinfection [55,56]. Last but not least, adaptive cellular immunity that underpins protection against severe disease [57] was not a subject of this study.

## 5. Conclusions

This study showed better anti-N and anti-RBD antibody response to SARS-CoV-2 infection in individuals vaccinated against seasonal influenza than in those who did not receive such vaccination. Further research is required to understand the mechanisms underlying this phenomenon. Nevertheless, the results add to accumulating evidence on the broadly beneficial effects of influenza vaccination in COVID-19.

## Figures and Tables

**Table 1:** The demographic characteristics of the studied groups of COVID-19 convalescent patients.

Parameter	Unvaccinated against Influenza (*n* = 292)	Vaccinated against Influenza (*n* = 489)	*p*-Value
Age (years), mean ± SD	35.8 ± 8.5	37.0 ± 10.3	>0.05
≥50 years, % (*n*)	5.1 (15)	11.9 (58)	0.002
Women/men, % (*n*)	17.1 (50)/82.9 (242)	23.3 (114)/76.7 (375)	>0.05
Comorbidities, % (*n*)	1.7 (5)	5.1 (25)	0.02

**Table 2:** The frequencies (%) of IgG antibodies against SARS-CoV-2 nucleocapsid protein (anti-N), receptor binding domain of spike protein (anti-RBD), subunit S2 of spike protein (anti-S2), and envelope protein (anti-E) in mild COVID-19 convalescent individuals not vaccinated and vaccinated against seasonal influenza. The *p*-value refers to difference between these groups examined with Pearson’s χ^2^ test.

IgG Antibodies	Unvaccinated against Influenza (*n* = 292)	Vaccinated against Influenza (*n* = 489)	*p*-Value	Total (*n* = 781)
anti-N	65.8	74.6	0.008	71.3
anti-RBD	76.7	85.1	0.001	81.6
anti-S2	39.7	42.9	>0.05	41.7
anti-E	1.7	1.4	>0.05	1.5

**Table 3:** Relationship (given as Spearman’s rank correlation coefficient) between serum concentrations of IgG antibodies against SARS-CoV-2 nucleocapsid protein (anti-N), receptor binding domain of spike protein (anti-RBD), subunit S2 of spike protein (anti-S2), and envelope protein (anti-E) in mild COVID-19 convalescent individuals not vaccinated and vaccinated against seasonal influenza.

IgG Antibodies	Unvaccinated against Influenza (*n* = 292)				Vaccinated against Influenza (*n* = 489)			
	anti-N	anti-RBD	anti-S2	anti-E	anti-N	anti-RBD	anti-S2	anti-E
anti-N	-	0.56	0.24	0.15	-	0.38	0.21	0.14
		***p* < 0.05**	***p* < 0.05**	*p* > 0.05		***p* < 0.05**	***p* < 0.05**	*p* > 0.05
anti-RBD	-	-	0.19	0.67	-	-	0.38	0.32
			*p* > 0.05	*p* > 0.05			***p* < 0.05**	*p* > 0.05
anti-S2	-	-	-	0.32	-	-	-	0.04
				*p* > 0.05				*p* > 0.05
anti-E	-	-	-	-	-	-	-	-

## Data Availability

The data presented in this study are available from the corresponding author upon reasonable request.
